# Optimizing Surveillance Performance of Alpha-Fetoprotein by Selection of Proper Target Population in Chronic Hepatitis B

**DOI:** 10.1371/journal.pone.0168189

**Published:** 2016-12-20

**Authors:** Jung Wha Chung, Beom Hee Kim, Chung Seop Lee, Gi Hyun Kim, Hyung Rae Sohn, Bo Young Min, Joon Chang Song, Hyun Kyung Park, Eun Sun Jang, Hyuk Yoon, Jaihwan Kim, Cheol Min Shin, Young Soo Park, Jin-Hyeok Hwang, Sook-Hyang Jeong, Nayoung Kim, Dong Ho Lee, Jaebong Lee, Soyeon Ahn, Jin-Wook Kim

**Affiliations:** 1 Department of Medicine, Seoul National University Bundang Hospital, Seongnam, Republic of Korea; 2 Department of Internal Medicine, Seoul National University College of Medicine, Seoul, Republic of Korea; 3 Division of Statistics, Medical Research Collaborating Center, Seoul National University Bundang Hospital, Seongnam, Republic of Korea; Osaka University Graduate School of Medicine, JAPAN

## Abstract

Although alpha-fetoprotein (AFP) is the most widely used biomarker in hepatocellular carcinoma (HCC) surveillance, disease activity may also increase AFP levels in chronic hepatitis B (CHB). Since nucleos(t)ide analog (NA) therapy may reduce not only HBV viral loads and transaminase levels but also the falsely elevated AFP levels in CHB, we tried to determine whether exposure to NA therapy influences AFP performance and whether selective application can optimize the performance of AFP testing in CHB during HCC surveillance. A retrospective cohort of 6,453 CHB patients who received HCC surveillance was constructed from the electronic clinical data warehouse. Covariates of AFP elevation were determined from 53,137 AFP measurements, and covariate-specific receiver operating characteristics regression analysis revealed that albumin levels and exposure to NA therapy were independent determinants of AFP performance. C statistics were largest in patients with albumin levels ≥ 3.7 g/dL who were followed without NA therapy during study period, whereas AFP performance was poorest when tested in patients with NA therapy during study and albumin levels were < 3.7 g/dL (difference in C statics = 0.35, p < 0.0001). Contrary to expectation, CHB patients with current or recent exposure to NA therapy showed poorer performance of AFP during HCC surveillance. Combination of concomitant albumin levels and status of NA therapy can identify subgroup of CHB patients who will show optimized AFP performance.

## Introduction

Hepatocellular carcinoma (HCC) is the fifth most common cancer in men ant the seventh in women worldwide [1]. The mortality of HCC is high, making it the third most common cause of cancer-related death [1]. Since tumour stage is one of the most important prognostic factors [2], early detection of HCC by surveillance may reduce cancer-related mortality in high-risk patients [3, 4]. Serum alpha-fetoprotein (AFP) testing has been the most commonly used method for HCC surveillance [5]. Recently, the usefulness of AFP in HCC surveillance has been challenged due to suboptimal sensitivity (50–60% at the cut-off of 20 ng/mL) [6–8]. Moreover, serum AFP levels may elevate in chronic hepatitis C virus infection without evidence of HCC [9–11].

Hepatitis B virus (HBV) infection is the leading cause of HCC worldwide [12], and HCC surveillance is recommended in patients with chronic HBV infection [13–15]. However, reported sensitivity and specificity of AFP varied widely in chronic hepatitis B (CHB) patients: 33–72% and 35–100%, respectively, at the cut-off of 20 ng/mL in recent literatures [16–20]. Similar to chronic hepatitis C virus infection, hepatitis activity and fibrosis stage may influence AFP levels in CHB [21–24]. To make matters more complicated, potent oral nucleos(t)ide analog (NA) therapy may reduce the elevated AFP levels [16, 23, 25, 26], and several reports suggested progressively or persistently elevated AFP as a specific marker for HCC in CHB patients on NA therapy [20, 23, 26].

If NA can improve specificity of AFP in a predictable way in CHB, then it would be theoretically possible to limit AFP testing to patients who are expected to show optimal test performance. Although determinants of AFP elevation have been well defined, it has not been proven whether viral replication and oral NA therapy significantly modify overall test performance of AFP in HBV-associated HCC surveillance.

In this study, we tried to determine whether NA therapy modifies overall AFP performance, and whether selective application can optimize the performance of AFP testing in CHB during HCC surveillance. To achieve these goals, we performed covariate-specific receiver operating characteristics regression analysis to determine covariates which independently influence the performance of AFP, and compared C statistics of AFP according to the covariates to identify subgroup(s) with optimized AFP performance among CHB patients on HCC surveillance.

## Materials and Methods

### Study design and population

This single centre retrospective cohort study recruited CHB patients from a structured chronic liver disease database that has been maintained since 2003 as a part of the electronic medical record system developed by our hospital (BESTCare) [27]. All consecutive CHB patients aged over 18 years with or without liver cirrhosis who received HCC surveillance between May 2003 and October 2015 were retrieved from the database. The following patients were excluded from the study cohort: 1) total surveillance period being less than 6 months, 2) diagnosis of HCC before or within 6 months after first surveillance examinations, and 3) hepatitis C virus or human immunodeficiency virus coinfection.

The surveillance program consisted of both abdominal ultrasonography (US) and serum AFP at 6 month intervals [28]. If the AFP level was higher than 20 ng/mL or increasing compared with previous measurements, the AFP measurement was repeated in 1–3 months. A dynamic imaging study (CT or MRI) was performed if 1) > 2 cm nodule(s) was detected that had not been previously characterized or that grew significantly compared to previous imaging studies or 2) serially measured AFP levels increased progressively. The diagnosis of liver cirrhosis was made based on biopsies or on the combination of clinical, ultrasonographic and endoscopic findings [7]. The diagnosis of HCC was based on biopsy or on typical enhancing patterns (hypervascular in the arterial phase with washout in the portal venous or delayed phases) by 2 techniques of dynamic imaging studies [14].

Oral NA therapy was initiated or maintained according to the 2008 Asian Pacific Association for the Study of the Liver guidelines which is in accordance with reimbursement policy of Korean national health insurance system: alanine aminotransferase [ALT] > 2 times upper limit of normal and HBV-DNA > 20,000 IU/mL if HBeAg-positive or > 2,000 IU/mL if HBeAg-negative [29]. Status of antiviral therapy in each patient was categorized as either exposure or non-exposure to oral NA therapy during study period. In the covariate-specific ROC regression analysis (described below), each AFP measurement was regarded associated with NA therapy if NA was prescribed within 1 year from the measurement date.

The institutional review board and ethics committee of Seoul National University Bundang Hospital approved this study (IRB No: B-1311/228-104). All clinical investigation has been conducted according to the principles expressed in the Declaration of Helsinki. Informed consent was wavered by IRB, due to the retrospective observational nature of study and anonymous analysis of data.

### Statistical analysis

All consecutive AFP values were analysed along with concomitantly measured biochemical, hematologic and virologic data. Biochemical and hematologic parameters were linked to each AFP value if measured within 7 days, and virologic parameters were linked if measured within 30 days from each AFP measurement. Each AFP value and related parameters for AFP change were assigned as case (HCC-associated measurement) if measured within 2 months before final diagnosis of HCC, and assigned as control (no HCC) if measured more than 6 months before final diagnosis of HCC or measured in patients without HCC development during study period. AFP values measured between 2 and 6 months before final diagnosis of HCC were assigned as “indeterminate” and excluded from analysis. AFP values measured more than 1 month after final diagnosis of HCC were also excluded.

All of statistical analyses were performed using STATA version 14 (College Station, Texas). Differences in the baseline characteristics between the patients with or without HCC were analysed using Student’s *t*–test or Mann–Whitney rank sum test for continuous variables and the χ^2^ test for categorical variables. Logistic regression analysis was performed to determine the factors associated with elevated AFP levels. Kaplan-Meier analysis with log rank test was used to estimate and compare the probability of HCC development during surveillance.

ROC analysis was used to assess the surveillance performance of AFP and other parameters. C statistics were compared by using “roccomp” command in order to determine whether NA therapy and other covariates of AFP elevation had an impact on the surveillance performance of AFP [30]. Effects of AFP covariates on AFP performance were simultaneously examined by covariate-specific ROC regression analysis using “rocreg” command with “roccov” options and 1000 bootstrap replications [31]. Briefly, the ROC curve is estimated as a cumulative distribution function *g* invoked with input of a linear polynomial of corresponding quantile function invoked on the false-positive rate *u*:

$$\text{ROC}(u)=g\{\text{X}´\beta +\alpha g^{-1}(u)\}$$

where the constant intercept *β* may depend on covariate and was tested for statistical inference.

## Results

### Baseline characteristics of study cohort and development of HCC

**Fig 1:**
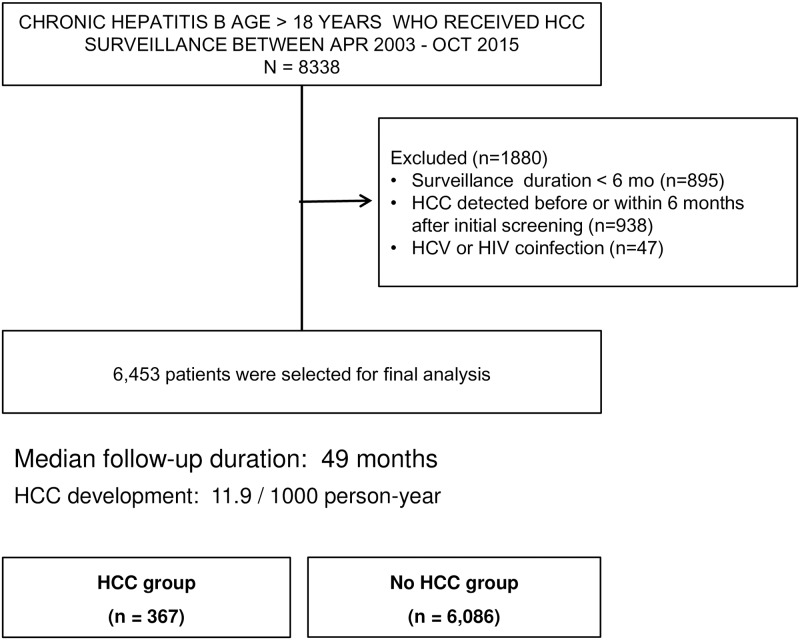
Flow of patient selection.

A total of 8,338 patients with chronic hepatitis B were identified from the BESTCare database. After excluding patients with incomplete surveillance data, HCC diagnosed within 6 months of surveillance or other viral coinfection, 6,453 patients were finally included in the cohort (Fig 1). As for antiviral treatment, 2981 patients (46%) received NAs, whereas 3472 patients were followed without NA therapy during study period. The types of NAs were presented in S1 Table.

**Fig 2:**
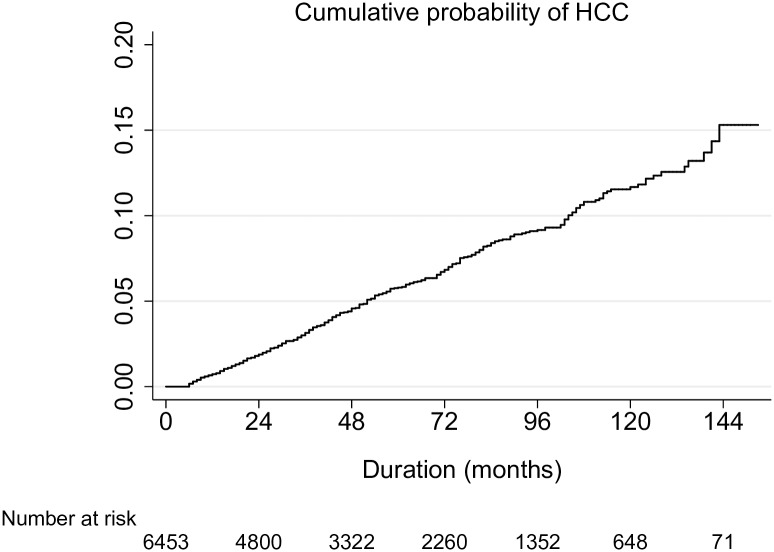
Incidence of hepatocellular carcinoma in the study cohort. Kaplan-Meier analysis showed that the cumulative risk of HCC was 0.7, 3.1, 5.8, 11.7% at 1, 3, 5, 10 years, respectively.

During the surveillance period, HCC was detected in 367 patients, with cumulative incidence of 11.9 per 1,000 person-years during median follow-up of 49 months (Fig 2). Characteristics of the HCC were summarized in S2 Table. Baseline characteristics of the cohort showed that patients who developed HCC during surveillance had older age, more male predominance, more cirrhosis, higher proportion of antiviral therapy, poorer hepatic function, higher HBV DNA and higher baseline AFP levels compared to patients who did not develop HCC (Table 1). Cox proportional hazards analysis confirmed that old age, male sex, prolonged prothrombin time, low platelet counts, high HBV DNA levels and elevated baseline AFP level were independent risk factors for HCC development (S3 Table).

**Table 1:** Baseline clinical and laboratory parameters in patients with or without subsequent HCC development during the surveillance period.

Parameters	All patients (n = 6,453)	HCC group (n = 367)	No HCC group (n = 6,086)	*P* value*
Age (year)	46 ± 12	54 ± 10	46 ± 12	< 0.0001
Gender, male (%)	3,814 (59)	261 (72)	3,553 (58)	< 0.0001
Liver cirrhosis (%)	1,342 (21)	294 (80)	1,048 (17)	< 0.0001
Nucleos(t)ide analog therapy (%)	2981 (46)	312 (85)	2669 (44)	< 0.0001
AST (IU/L)	30 (7–4898)	50 (13–449)	29 (7–4898)	0.0816
ALT (IU/L)	34 (3–6824)	49 (12–1256)	33 (3–6824)	0.8682
Albumin (mg/dL)	4.2 ± 0.4	3.8 ± 0.5	4.3 ± 0.4	< 0.0001
Bilirubin (mg/dL)	0.9 (0.2–24.2)	1.1 (0.2–14.6)	0.9 (0.2–24.2)	< 0.0001
Prothrombin time (INR)	1.08 ± 0.18	1.19 ± 0.18	1.07 ± 0.17	< 0.0001
Platelet count (x1000 /mm^3^)	191 ± 66	126 ± 55	195 ± 64	< 0.001
HBV DNA (IU/mL)	5,124 (25–30,400,000)	385,864 (25–30,300,000)	3864 (25–30,400,000)	0.0183
AFP (ng/mL)	3.0 (0.1–4000)	8.0 (0.3–4000)	3.0 (0.1–3573)	< 0.001
AFP > 10 ng/mL	782 (12)	148 (40)	634 (10)	< 0.001

*HCC vs. no HCC group

Continuous values are expressed as mean ± standard deviation (SD) or median (range), and categorical values are expressed as number (%).

### Determinants of AFP levels during HCC surveillance

A total of 53,343AFP measurements were made during study period, with median of 6 AFP measurements per patient. After excluding 206 indeterminate measurements as defined in Methods, 53,137 AFP values were finally used for analysis. Plotting of serial AFP values showed variant nature of AFP levels during surveillance (S1 Fig).

When binomial logistic regression analysis was performed using the 53,137 AFP measurements as dependent variable (≤ 10 ng/mL vs. > 10 ng/mL), the following factors were independently associated with elevated AFP levels during surveillance: development of HCC, presence of liver cirrhosis, elevated transaminase levels, decreased albumin levels, prolonged prothrombin time, thrombocytopenia, high HBV viral loads and status of NA treatment (Table 2). Patient with detectable serum HBV DNA had 3.8 times higher odds of AFP elevation. Patients who ever received oral NA during study period had 2.0 times higher odds of AFP elevation compared patients without NA therapy during surveillance.

**Table 2:** Multivariate logistic regression model for covariates of elevated AFP levels (> 10 ng/mL) during HCC surveillance.

Parameter	Odds ratio	95% C.I.	P value
HCC			
No vs. Yes	18.1	13.0–25.3	< 0.001
Liver cirrhosis			
No vs. Yes	1.62	1.39–1.91	< 0.001
AST (IU/L)			
≤ 60 vs. >60	3.52	2.90–4.27	< 0.001
ALT (IU/L)			
≤ 60 vs. >60	2.18	1.80–2.64	< 0.001
Albumin (g/dL)			
≥ 3.7 vs. < 3.7	3.69	3.07–4.44	< 0.001
Bilirubin (mg/dL)			
≤ 1.5 vs. > 1.5	0.95	0.77–1.17	0.635
PT (INR)			
≤ 1.1 vs. >1.1	2.22	1.90–2.59	< 0.001
Platelet count			
≥120K vs < 120K/mm^3^	1.21	1.02–1.44	0.027
HBV DNA			
≤ 60 vs. > 60 IU/mL	3.99	3.35–4.75	< 0.001
NA therapy			
No vs. Yes	2.02	1.68–2.45	< 0.001

### Determinants of AFP performance during HCC surveillance: effects of NA therapy and other covariates of increased AFP levels

After confirming independent covariates of AFP elevation, we examined the influence of these covariates on the performance of AFP by comparing C statistics. The overall sensitivity and specificity of AFP were 54.3% and 93.5%, respectively, at the cut-off of 10 ng/mL, and 44.5% and 96.6%, respectively, at the cut-off of 20 ng/mL. Univariate ROC analysis identified covariates which affected surveillance performance of AFP: the c-statistics were lower in patients with cirrhosis, elevated AST levels, low albumin levels, prolonged prothrombin time, elevated baseline AFP levels, and exposure to NA therapy during study period (Table 3).

**Table 3:** Comparison of C statistics of AFP measurements according to covariates of AFP elevation and baseline AFP levels.

Parameter	C statistic	95% C.I.	P value
Liver cirrhosis			
No	0.872	0.8284–0.916	
Yes	0.766	0.737–0.794	< 0.001
AST (IU/L)			
≤ 60	0.829	0.804–0.855	
> 60	0.764	0.709–0.820	0.038
ALT (IU/L)			
≤ 60	0.837	0.812–0.861	
> 60	0.796	0.739–0.853	0.195
Albumin (g/dL)			
≥ 3.7	0.825	0.7956–0.856	
< 3.7	0.726	0.686–0.766	< 0.001
PT (INR)			
≤ 1.1	0.844	0.801–0.888	
>1.1	0.776	0.741–0.811	0.018
HBV DNA			
≤ 60 IU/mL	0.794	0.746–0.843	
> 60 IU/mL	0.848	0.814–0.881	0.074
NA therapy			
No	0.889	0.856–0.921	
Yes	0.800	0.770–0.830	< 0.001
Baseline AFP			
≤ 10 ng/mL	0.825	0.794–0.856	
> 10 ng/mL	0.763	0.726–0.801	0.012

Next, multivariate regression analysis of ROC was performed to identify independent covariates of AFP performance. Among the significant covariates in Table 3, low concomitantly measured albumin levels and status of NA therapy were independently associated with reduced C statistics of AFP (Table 4): AFP performance was poorer in patients on maintenance NA therapy or with exposure history within 1 year from AFP measurement. The impact of NA therapy on the C statistics retained significance in AFP values associated with HBV DNA > 200 IU/mL, but lost significance when analysis was limited to AFP measurements associated with HBV DNA levels ≤ 200 IU/mL (S2 Fig).

**Table 4:** Covariate-adjusted ROC regression analysis of AFP for HCC surveillance.

Covariate	β*	95% CI (lower, upper bound)**
Liver cirrhosis (No vs. Yes)	0.028	-0.406, 0.462
AST (≤ 60 vs. > 60 IU/L)	0.009	-0.471, 0.489
Albumin (≥ 3.7 vs. < 3.7 g/dL)	**-0.639**	**-1.273, - 0.048**
PT (INR) (≤ 1.1 vs. >1.1)	-0.368	-0.844, 0.108
HBV DNA (≤ 60 vs. > 60 IU/mL)	-0.196	-0.561, 0.169
NA therapy***	**-0.431**	**-0.848, -0.014**
Baseline AFP (≤ 10 vs. > 10 ng/mL)	-0.121	-0.553, 0.311

*Negative sign indicates detrimental effect on C statistics.

**Inferred from 1000 bootstrap replications; inclusion of 0 indicates statistically insignificant effect of covariate on the ROC curve.

*** Exposure history to NA within 1 year from AFP measurement

### Comparison of AFP performance according to concomitant albumin levels and NA therapy status: identification of conditions in which AFP works best or worst

According to the result of multivariate ROC regression analysis, all AFP measurements were classified into 2x2 groups by concomitant albumin levels and NA therapy status for sensitivity and specificity analysis (Table 5). The Youden index was negatively affected by NA treatment and low albumin levels. These two factors were associated with lower sensitivity at a similar specificity.

**Table 5:** Sensitivity and specificity of AFP according to concomitant albumin levels and NA therapy.

Group	1	2	3	4
	NA (-) Albumin ≥ 3.7	NA (+) Albumin ≥ 3.7	NA (-) Albumin < 3.7	NA (+) Albumin < 3.7
Number (HCC-associated)	20,702 (105)	29,330 (234)	685 (37)	2,420 (73)
Youden index	0.646	0.497	0.369	0.215
Cutoff (ng/mL)	3.9	5.3	25.0	67.2
Sensitivity	80	65	49	33
Specificity	85	85	87	89

**Fig 3:**
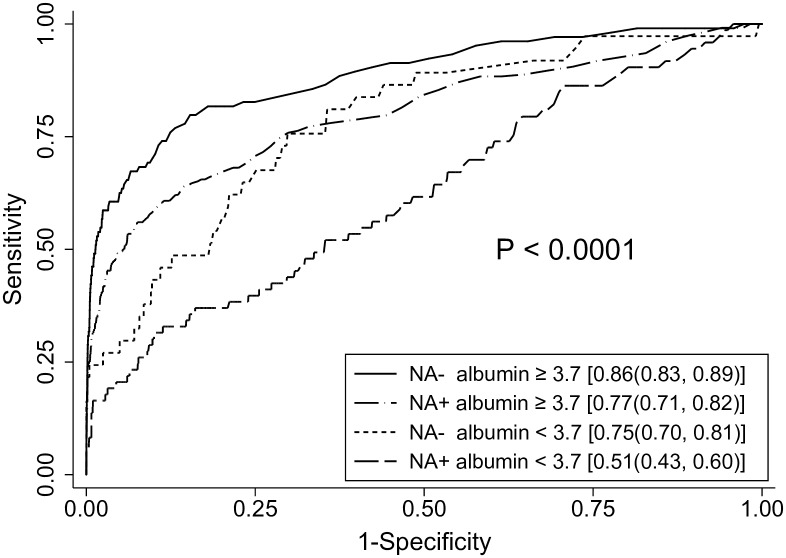
Receiver operating characteristic curves of AFP according to NA therapy status and concomitant albumin levels. The C statistics with 95% confidence interval are presented in brackets. AFP tests showed best performance in patients without NA therapy during study period and when concomitantly measured albumin levels were ≥ 3.7 g/dL. In contrast, the C statistics were lowest in patients who received NA therapy during study period and when concomitantly measured albumin levels were < 3.7 g/dL.

The ROC analysis of AFP showed that C statistics were largest when AFP testing was performed in patients without NA therapy during study period and when concomitantly measured albumin levels were ≥ 3.7 g/dL, whereas AFP performance was poorest when tested in patients who received NA therapy during study period and when concomitantly measured albumin levels were < 3.7 g/dL (difference in C statics = 0.35, p < 0.0001; Fig 3).

## Discussion

The results of this study confirmed the hypothesis that the surveillance performance of AFP depends on disease severity (serum albumin levels) and exposure history to oral antiviral therapy in CHB, and that it is possible to define subgroups with significantly different C statistics according to the two covariates. Recent European and American practice guidelines do not recommend use of AFP in HCC surveillance due to its suboptimal performance [14, 32]. However, host and viral factors were suggested to influence AFP performance [33, 34], so that there is possibility that tailored application of testing may improve overall AFP performance [34]. To the best of our knowledge, this is the first large-scale cohort study which identified the determinants of AFP performance by ROC regression analysis, and defined optimized conditions for AFP-based surveillance in CHB. Surprisingly, however, exposure to NA therapy was associated with poorer performance of AFP.

One of the methodological strengths of this study is analysis of all consecutive AFP measurements from each patient instead of single representative AFP values, which might introduce bias because AFP levels frequently fluctuate in chronic viral hepatitis patients with hepatitis flare-up, hepatic fibrosis and hepatic dysfunction (S1 Fig) [9, 10, 21, 24, 35, 36]. More importantly, matching of all serial AFP values with concomitantly measured covariates allowed identification of covariates for AFP elevation and for AFP performance in the whole surveillance periods.

Multivariate analysis for AFP elevation *during surveillance* revealed that, in addition to previously known covariates of AFP, serum HBV DNA levels and status of oral NA therapy were also independent predictors (Table 2). The finding that viral load independently predicted AFP elevation may suggest direct interaction between HBV replication and AFP expression in HCC cells, in addition to hepatitis activity induced by HBV. This hypothesis may also explain decreased sensitivity of AFP in patients who received NA therapy (below), but further validation is needed. It may seem contradictory that maintenance NA therapy, which will suppress HBV DNA, was also associated with AFP elevation. We speculated that disease stage itself (i.e., immune clearance phase of CHB in which NA therapy is indicated) might have greater effect on AFP levels than antiviral action of NA had. However, this hypothesis also warrants further studies.

Most covariates of AFP elevation except for ALT and HBV DNA had deleterious effect on the AFP C statistics by univariate ROC analysis (Table 3). Although HBV DNA was a significant covariate of AFP elevation, it had no significant impact on the C statistics of AFP. This is understandable because covariates which affect the distribution of marker (i.e. AFP) in the control (i.e. no HCC) may or may not impact the separation between cases (i.e. HCC) and controls [31].

Multivariate ROC regression analysis identified low albumin levels and exposure history to NA therapy as independent determinants for AFP performance during HCC surveillance (Table 4). It is interesting that serum albumin level was the only significant laboratory parameter. We believe that transaminase levels were excluded in the final multivariate model because ALT elevation was a condition of NA therapy, and that other markers of disease severity were replaced by albumin. Hypoalbuminemia contribution to impaired AFP performance needs further explanatory studies.

Contrary to the previous expectations that use of NA may help improve the test performance of AFP [16, 20, 23], rigorous comparisons of C statistics revealed that AFP performance deteriorated with exposure history to NA therapy. The main reason for the deterioration was apparently due to decreased sensitivity (Table 5). It can be speculated that HBV replication might directly induce AFP expression in HCC cells, and NAs suppress expression of AFP in HCC leading to decreased sensitivity of AFP. Another possible hypothesis is that disease stage (immune clearance phase) rather than NA therapy itself might be associated with poorer performance of AFP. Both hypotheses might explain our multivariate regression data, so that the mechanistic explanations need to be provided by further studies. In either case, our results clearly demonstrated limited role of AFP in detecting HCC in CHB patients with current or recent exposure to NA therapy.

Finally, we classified AFP measurements according to concomitant albumin levels and NA therapy status, and compared C statistics between the subgroups. Indeed, the two parameters were able to identify subgroups with significantly different AFP performance (difference between best and worst C statistics was 0.35, p < 0.0001; Fig 3). This finding suggests that it would be possible to optimize the AFP performance by tailored application of testing according to the pre-defined predictors.

There are limitations to this study. Because this was a retrospective cohort study from a single institute, prospective validation is needed to confirm the efficacy of tailored AFP testing. Second, this study was designed to determine the effects of covariates on AFP performance, so that we did not attempt to prove the ultimate usefulness of AFP (i.e., survival benefit) as a surveillance tool. Finally, the effect of oral NAs on AFP performance should be further explored because the link between oral NAs and AFP performance was associative, not causal.

In conclusion, performance of AFP is dependent on concomitant albumin levels and status of NA therapy during surveillance for HBV-related HCC. Contrary to prior expectation, CHB patients with current or recent exposure to NA therapy showed poorer performance of AFP compared to patients without NA therapy. Combination of concomitant albumin levels and status of NA therapy can identify subgroup of CHB patients who will show optimized AFP performance.

## Supporting Information

S1 Fig Serum AFP profiles during HCC surveillance. The upper and lower panel indicates serial plots of AFP measurements from each patient with or without subsequent development of HCC, respectively. In the HCC group, the AFP levels after the diagnosis of HCC were included to show the progressive trends.
(TIF)

S2 Fig Effect of nucleos(t)ide analog therapy on C statistics of AFP according to concomitant HBV viral loads. Patients without NA therapy had highest C statistics compared to patients exposed to NA treatment during study period (left panel). Subgroup analysis showed that the effect of NA was significant when concomitant HBV DNA levels were > 200 IU/mL (right panel), whereas status of NA therapy did not affect C statistics of AFP when HBV DNA levels were ≤ 200 IU/mL (central panel). C statistics are in parentheses.
(TIF)

S1 Table Nucleos(t)ide analogues used in the study population. (DOCX)

S2 Table Characteristics of HCC detected during surveillance (N = 367). (DOCX)

S3 Table Baseline predictors of hepatocellular carcinoma by Cox proportional hazard analysis. (DOCX)
