# Supplementary figures and images for: Optimizing Surveillance Performance of Alpha-Fetoprotein by Selection of Proper Target Population in Chronic Hepatitis B
Source: PLoS One. 2016 Dec 20;11(12):e0168189. doi: 10.1371/journal.pone.0168189 (PMC5172583; doi:10.1371/journal.pone.0168189)

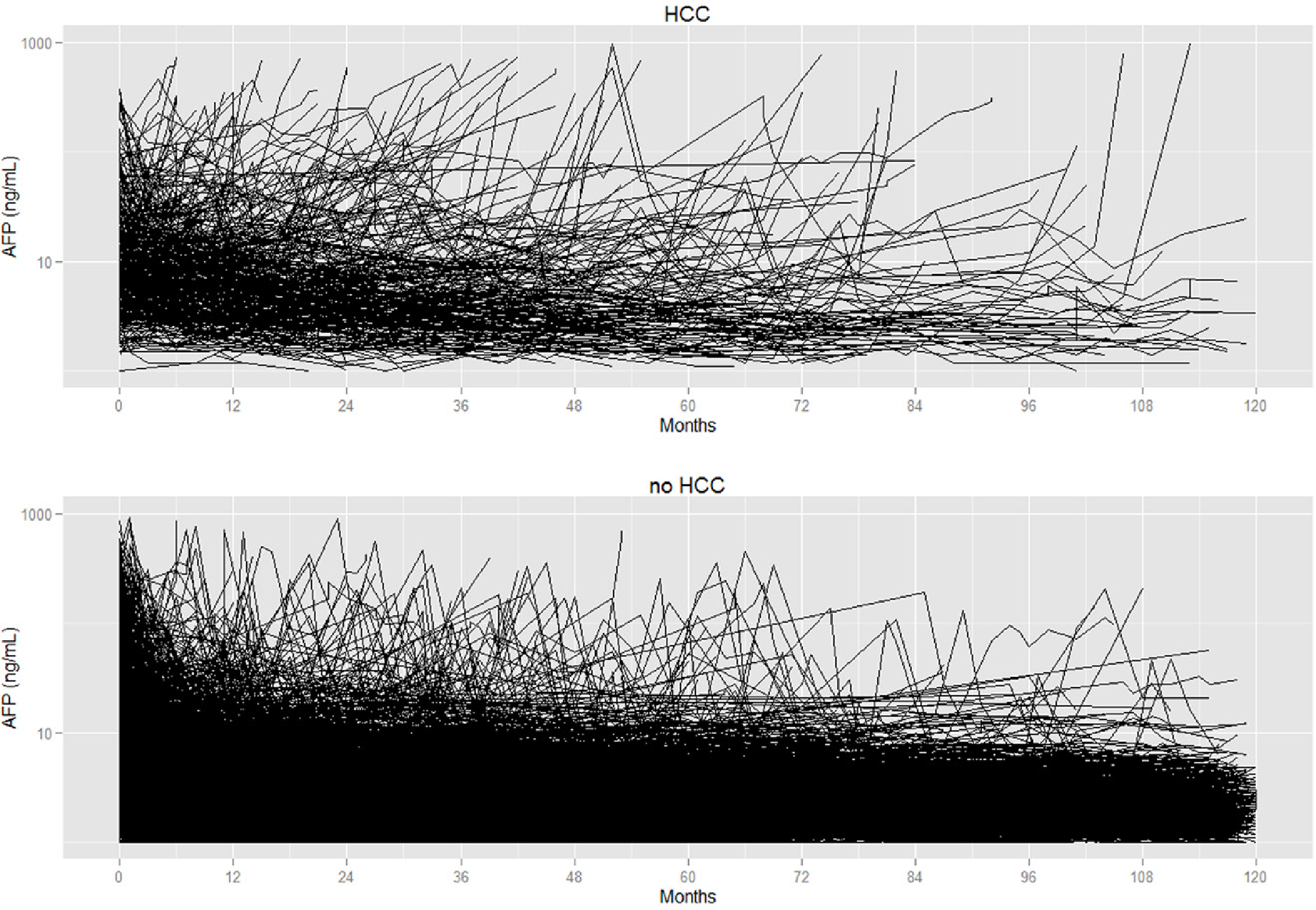

Supplement: S1 Fig — The upper and lower panel indicates serial plots of AFP measurements from each patient with or without subsequent development of HCC, respectively. In the HCC group, the AFP levels after the diagnosis of HCC were included to show the progressive trends. (TIF) [file pone.0168189.s001.tif]

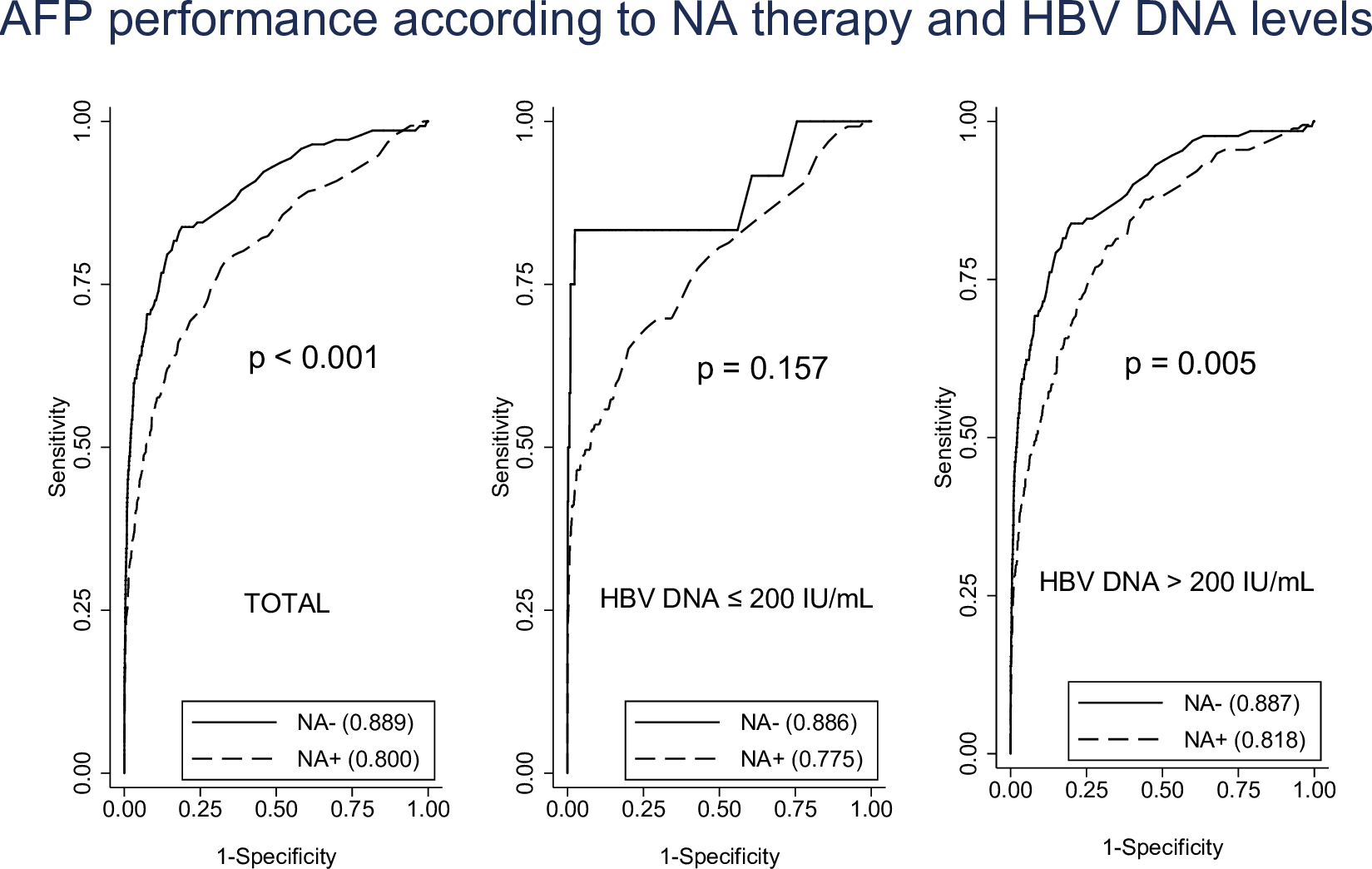

Supplement: S2 Fig — Patients without NA therapy had highest C statistics compared to patients exposed to NA treatment during study period (left panel). Subgroup analysis showed that the effect of NA was significant when concomitant HBV DNA levels were > 200 IU/mL (right panel), whereas status of NA therapy did not affect C statistics of AFP when HBV DNA levels were ≤ 200 IU/mL (central panel). C statistics are in parentheses. (TIF) [file pone.0168189.s002.tif]
