# Supplementary material for: Optimizing Surveillance Performance of Alpha-Fetoprotein by Selection of Proper Target Population in Chronic Hepatitis B
Source: PLoS One. 2016 Dec 20;11(12):e0168189. doi: 10.1371/journal.pone.0168189 (PMC5172583; doi:10.1371/journal.pone.0168189)
Supplement: S1 Table — (DOCX) [file pone.0168189.s003.docx]

**Supplementary Data**

**Optimization of alpha-fetoprotein performance by utility score in chronic hepatitis B**

Jung Wha Chung^1^*,* Beom Hee Kim^1^, Chung Seop Lee^1^, Gi Hyun Kim^1^, Hyung Rae Sohn^1^, Bo Young Min^1^, Joon Chang Song^1^, Hyun Kyung Park^1^, Eun Sun Jang^1^, Hyuk Yoon^1^, Jaihwan Kim ^1^, Cheol Min Shin ^1^, Young Soo Park^1,2^, Jin-Hyeok Hwang ^1,2^, Sook-Hyang Jeong^1,2^, Nayoung Kim^1,2^, Dong Ho Lee^1,2^, Jaebong Lee^3^, Soyeon Ahn^3^, and Jin-Wook Kim^1,2^

**Table of contents**

Supplementary tables: Table S1, Table S2, Table S3

**Supplementary tables**

**S1Table. Nucleos(t)ide analogues used in the study population**

| Nucleos(t)ide analogue | N | (%) |
| --- | --- | --- |
| Adefovir | 686 | 14 |
| Clevudine | 211 | 4 |
| Entecavir | 1982 | 41 |
| Lamivudine | 1156 | 24 |
| Telbivudine | 75 | 2 |
| Tenofovir | 707 | 15 |

Some patients received more than one NA, either sequentially or in combinaiton.
