# Supplementary material for: Optimizing Surveillance Performance of Alpha-Fetoprotein by Selection of Proper Target Population in Chronic Hepatitis B
Source: PLoS One. 2016 Dec 20;11(12):e0168189. doi: 10.1371/journal.pone.0168189 (PMC5172583; doi:10.1371/journal.pone.0168189)
Supplement: S3 Table — (DOCX) [file pone.0168189.s005.docx]

**Supplementary Data**

**Optimization of alpha-fetoprotein performance by utility score in chronic hepatitis B**

Jung Wha Chung^1^*,* Beom Hee Kim^1^, Chung Seop Lee^1^, Gi Hyun Kim^1^, Hyung Rae Sohn^1^, Bo Young Min^1^, Joon Chang Song^1^, Hyun Kyung Park^1^, Eun Sun Jang^1^, Hyuk Yoon^1^, Jaihwan Kim ^1^, Cheol Min Shin ^1^, Young Soo Park^1,2^, Jin-Hyeok Hwang ^1,2^, Sook-Hyang Jeong^1,2^, Nayoung Kim^1,2^, Dong Ho Lee^1,2^, Jaebong Lee^3^, Soyeon Ahn^3^, and Jin-Wook Kim^1,2^

**Table of contents**

Supplementary tables: Table S1, Table S2, Table S3

**S3 Table. Baseline** predictors of hepatocellular carcinoma by Cox **proportional hazard analysis.**

| Covariate | Hazard ratio | 95% Confidence interval | P value |
| --- | --- | --- | --- |
| Age > 50 | 2.93 | 2.33 - 3.67 | < 0.001 |
| Male sex | 1.63 | 1.29 - 2.06 | < 0.001 |
| GOT > 60 IU/L | 1.70 | 1.20 - 2.41 | 0.003 |
| GPT > 60 IU/L | 1.17 | 0.81 - 1.70 | 0.398 |
| Albumin < 3.7 g/dL | 1.34 | 0.99 - 1.82 | 0.056 |
| Bilirubin > 1.5 mg/dL | 1.09 | 0.81 - 1.49 | 0. 567 |
| Prothrombin time (INR) >1.1 | 1.32 | 1.01 - 1.73 | 0.039 |
| Platelet count < 120K /mm^3^) | 4.16 | 3.19 - 5.44 | < 0.001 |
| HBV DNA > 60 IU/mL | 2.09 | 1.67 - 2.61 | < 0.001 |
| AFP > 10 ng/mL | 2.39 | 1.91 - 3.00 | < 0.001 |
